# Visceral Fat Accumulation Is Associated with Colorectal Cancer in Postmenopausal Women

**DOI:** 10.1371/journal.pone.0110587

**Published:** 2014-11-17

**Authors:** Jee-Yon Lee, Hye-Sun Lee, Duk-Chul Lee, Sang-Hui Chu, Justin Y. Jeon, Nam-Kyu Kim, Ji-Won Lee

**Affiliations:** 1 Department of Family Medicine, Yonsei University, College of Medicine, Seodaemun-gu, Seoul, Republic of Korea; 2 Department of Biostatistics, Yonsei University, College of Medicine, Seodaemun-gu, Seoul, Republic of Korea; 3 Department of Clinical Nursing Science, Yonsei University, College of Nursing, Nursing Policy Research Institute, Biobehavioural Research Centre, Seodaemun-gu, Seoul, Republic of Korea; 4 Department of Sport and Leisure Studies, Sports Medicine Laboratory, Yonsei University, Seodaemun-gu, Seoul, Republic of Korea; 5 Department of General Surgery, Yonsei University College of Medicine, Seodaemun-gu, Seoul, Republic of Korea; Robert Gordon University, United Kingdom

## Abstract

**Background:**

Obesity is a known risk factor for colorectal cancer (CRC), and emerging data suggest that this association is mediated by visceral fat rather than total body fat. However, there is a lack of studies evaluating the association between visceral fat area and the prevalence of CRC.

**Methods:**

To investigate the relationship between visceral adiposity and prevalence of CRC, data of 497 women diagnosed with CRC and 318 apparently healthy women were analysed and data of well-balanced 191 pairs of women with CRC and healthy women matched based on propensity scores were additionally analysed. Diagnosis of CRC was confirmed by colonoscopy and histology. Metabolic parameters were assessed, along with body composition, using computed tomography.

**Results:**

The median visceral fat area was significantly higher in the CRC group compared with the control group before and after matching. The prevalence of CRC increased significantly with increasing visceral fat tertiles after matching (p for trend <0.01). A multivariate analysis showed that mean visceral fat area of individuals in the 67^th^ percentile or greater group was associated with an increased prevalence of CRC (adjusted odds ratio: 1.80; 95% confidence interval: 1.12–2.91 before matching and adjusted odds ratio: 2.96; 95% confidence interval: 1.38–6.33) compared with that of individuals in the 33^th^ percentile or lower group.

**Conclusion:**

Thus, we conclude that visceral fat area is positively associated with the prevalence of CRC. Although we could not determine the causality, visceral adiposity may be associated with the risk of CRC. Further prospective studies are required to determine the benefits of controlling visceral obesity for reducing CRC risk.

## Introduction

Obesity and cancer are emerging as two of the most serious health problems worldwide. Obesity is known to increase the risk of cardio-metabolic diseases including Type 2 diabetes mellitus (DM), cardiovascular disease, and metabolic syndrome [1], [2]. Furthermore, the relationship between obesity and several types of cancer such as renal, oesophageal, colorectal, and breast cancer has also been reported [3], [4]. The precise underlying mechanism that explains how obesity promotes these diseases is still unclear; however, recent evidence suggests that visceral adipose tissue may play a key role in this relationship. Visceral adipose tissue, largely distributed in the abdominal cavity, shows higher hormonal and metabolic activities than subcutaneous fat tissue [5]. Visceral adipocyte-secreted growth factors, proinflammatory cytokines, and adipokines are considered mediating factors associated with the carcinogenesis of obesity-related tumours [6].

Colorectal cancer (CRC) is well known as an ‘obesity-related’ cancer. Recent epidemiologic studies have shown that waist circumference or the waist-hip ratio, which reflect abdominal adiposity rather than total body mass index (BMI), showed greater association with increased risk of CRC [7]–[9]. These findings indicate that the regional distribution of adipose tissue, not overall adiposity, may contribute to the increased risk of CRC. Altered metabolic activity and systemic chronic inflammation induced by visceral adipose tissue are also considered to be related with colorectal carcinogenesis [10]. A few studies have assessed the relationship between CRC risk and visceral obesity using a direct method to measure visceral fat area; however, the results were inconclusive [11]–[13]. Some studies showed increased CRC risk with higher visceral adipose tissue accumulation. However, no significant relationship, and even opposing results, have been reported.

Therefore, we investigated the relationship between the prevalence of CRC and visceral fat area by comparing a colorectal cancer group and a case-matched control group of Korean women.

## Methods

### Ethical statement

All subjects participated in the study voluntarily, and written informed consent was obtained from each participant. The study complied with the Declaration of Helsinki, and the Institutional Review Board of Yonsei University College of Medicine approved this study.

### Study subjects

The study subjects consisted of 1920 postmenopausal women who visited the Department of Colorectal Surgery and were diagnosed with CRC during their visit and 670 postmenopausal women who visited the Health Promotion Centre and the Department of Family Medicine at Severance Hospital for routine health check-ups that included a screening colonoscopy between November 2010 and August 2012. Menopausal status was defined as having had no menstrual periods for 12 consecutive months without any biological or physiological cause. We excluded women who were taking medication for a diagnosis of hypertension, diabetes mellitus, chronic liver disease, chronic renal disease, coronary artery occlusive disease, or stroke. We also excluded women who underwent polyp removal procedures or who were diagnosed with CRC or other types of cancer prior to their participation in the study. After applying the exclusion criteria, a total of 497 women diagnosed with CRC were defined as the CRC group, and 318 apparently healthy women were defined as the control group. From the CRC and healthy groups, a well-balanced study population consisting of 199 pairs of women was selected by propensity score matching.

### Measurement of clinical parameters

All subjects completed a questionnaire about their lifestyle, such as smoking, alcohol consumption, regular exercise, underlying medical conditions, and medications. Cigarette smoking was defined as current or past smokers, and alcohol consumption was defined as drinking alcohol more frequently than once per week or more than 70 grams per week during the previous year.

Blood pressure was measured in the sitting position after the subject was asked to rest for longer than 10 minutes. The mean blood pressure (mmHg) was calculated using the systolic blood pressure (SBP) and diastolic blood pressure (DBP) as follows: (SBP+2XDBP)/3. Body mass index (BMI) was defined as weight (kg) divided by height squared (m^2^).

Blood samples were collected after at least 8 hours of fasting. Fasting glucose, aspartate aminotransferase (AST), alanine aminotransferase (ALT), creatinine, and total cholesterol levels were measured by using the Hitachi 7600 Automatic analyzer (High-Technologies Corporation, Hitachi, Tokyo, Japan). White blood cell (WBC) counts were measured using an automated blood cell counter (ADVIA 120, Bayer, NY, USA). The biomarkers were part of the routine tests for patients who were planning to receive CRC surgery. The control group also have received the same blood tests as a part of their routine health check-ups.

### Assessment of body composition

Abdominal fat tissue areas were measured by computed tomography (Tomoscan 350; Philips, Mahwah, NJ, USA) as described previously [14]. A single cross-sectional CT image of a 3-mm thick slice at the level of L4–L5 interspace was obtained with the subject in a supine position. The visceral and subcutaneous fat areas were calculated at this slice using a commercially available software program (TeraRecon Aquarius; TeraRecon, CA, USA), which determined the fat area electronically by setting the attenuation range from −150 to −50 Hounsfield units. Visceral adipose tissue areas were measured by delineating the intra-abdominal cavity at the internal aspect of the abdominal and oblique muscle walls surrounding the cavity and the posterior aspect of the vertebral body. The subcutaneous adipose tissue area was calculated by subtracting the VAT area from the total adipose tissue area. All measurements were performed by a skilled radiologist who was blinded to the patient data. The inter- and intra-coefficients of variation (CVs) for reproducibility were 1·4% and 0·5%, respectively.

### Diagnosis of CRC

All participants received colonoscopic examinations performed by experienced gastroenterologists after bowel preparation with 4 litres of polyethylene glycol solution (Colyte; Taejun, Seoul, Korea). All procedures were performed by using a standard video colonoscope (CFQ240L, Olympus, Optical, Tokyo, Japan). Biopsies were taken from all detected suspicious lesions, and the final diagnosis of CRC was made by histopathological analysis. CRC was diagnosed if malignant cells were observed above the muscularis mucosae. The classification system recommended by the American Joint Committee on Cancer (AJCC) was used for tumour staging [15]. The locations of the tumours were recorded and divided into sigmoid, ascending, transverse, and descending colon, and rectum.

### Statistical analyses

Data for demographic characteristics are represented as the mean ± standard deviation or number (%). To reduce the effect of confounding factors that may affect the relationship between CRC and visceral adiposity, we adjusted for differences in the clinical basal characteristics between the CRC and control groups using propensity score matching [16]. The demographic characteristics of the CRC and control groups before matching were compared using two-sample *t*-tests for continuous data and Chi-square tests or Fisher's exact tests for categorical data. All variables constituting baseline demographic characteristics, such as age, BMI, smoking status, alcohol consumption, and regular exercise, were included as exact matching factors. A propensity score for the predicted probability of cancer in each woman was estimated using a logistic regression model fit with five factors. The controls were matched 1∶1 with CRC patients. A nearest-neighbour-matching algorithm with a greedy heuristic was used to match patients for demographic characteristics. The matched demographic characteristics of the CRC and control groups were compared using paired *t*-tests for continuous data and McNemar tests for categorical data. The metabolic parameters were described as median and interquartile range, and differences between the two groups after matching were compared using Wilcoxon signed-rank tests.

Tertiles were categorized as follows based on visceral fat areas: Q1: <67.98 cm^2^, Q2: 67.98–91.67 cm^2^, Q3: >91.67 cm^2^. The prevalence of CRC according to the visceral fat tertiles was compared using the Cochran-Armitage trend test. The odds ratio and 95% confidence intervals (CI) for CRC were calculated using conditional logistic regression analyses after adjusting for confounding factors across visceral fat tertiles.

All statistical analyses were performed using SAS software version 9.2 (SAS Institute Inc., Cary, NC, USA).

## Results

### Characteristics of the study population

The clinical characteristics of the CRC and control groups before and after propensity score matching are given in Table 1. Women with CRC showed a significantly higher age and lower BMI, and lower incidence of regular exercise. After propensity score matching was completed, there were 199 matched pairs of participants. There were no significant differences in clinical characteristics between the two groups.

**Table 1:** Comparison of demographic characteristics between the control group and the colorectal cancer group before and after propensity score matching.

	Unmatched			Matched		
	Control (n = 318)	Cancer (n = 497)	*P*-value a	Control (n = 199)	Cancer (n = 199)	*P*-value b
Age	58.38±9.84	60.70±12.29	<0.01	60.73±8.55	60.73±8.55	>.99
BMI (kg/m2)	23.91±2.39	23.4±3.55	0.02	23.84±2.42	23.56±3.18	>.99
Smoking status (yes) (%)	11(3.46)	13(2.62)	0.49	2(1.01)	2(1.01)	>.99
Alcohol consumption (yes) (%)	21(6.60)	20(4.02)	0.10	2(1.01)	2(1.01)	>.99
Regular exercise (yes) (%)	82(25.79)	34(6.84)	<.001	9(4.52)	9(4.52)	>.99

Normality was tested by the Kolmogorov-Smirnov test.

Data are the mean± standard deviation or percentage.

a *P* - values were derived from an independent-sample *t*-test for continuous data, and Chi-square test was performed for categorical data.

b *P* - values were derived from a paired *t*-test for parametric data or the McNemar test for categorical data.

Abbreviation: BMI, body mass index.

Table 2 shows the metabolic parameters of the CRC and control groups before and after matching. Visceral fat area, visceral/subcutaneous fat ratio, mean blood pressure, fasting glucose levels, WBC count, and creatinine levels were significantly higher in the CRC group compared to the control group before and after matching (p<0.05). The subcutaneous fat area was significantly lower in the CRC group compared to the control group before and after matching (p<0.05). ALT levels were significantly higher in the control group only before matching (p<0.01)

**Table 2:** Comparison of metabolic parameters between the control group and the colorectal cancer group before and after propensity score matching.

	Unmatched			Matched		
	Control (n = 318)	Cancer (n = 497)	P-value a	Control (n = 199)	Cancer (n = 199)	P-value a
Visceral fat area (cm2)	74.02(24.58–246.04)	88.2(19.3–256.2)	<0.01	73.47(24.58–246.04)	87.2(19.3–231.3)	<0.01
Subcutaneous fat area (cm2)	209.4(48.09–437.09)	189.5(22.8–445.2)	<0.01	205.79(48.09–394.44)	195.1(81.4–445.2)	0.02
Visceral/Subcutaneous Fat ratio (%)	0.35(0.09–2.04)	0.45(0.14–1.72)	<0.01	0.34(0.09–2.04)	0.44(0.18–1.14)	<0.01
Mean blood pressure (mmHg) b	86.67(67–117)	89.33(65.67–136)	<0.01	86.67(67–117)	90.33(68.33–121.67)	<0.01
Fasting glucose (mg/dl)	90(64–170)	98(69–384)	<0.01	90(64–170)	100(69–326)	<0.01
Total cholesterol (mg/dl)	183(100–270)	182(76–333)	0.57	178(100–270)	188(80–285)	0.36
WBC (counts/L)	5475(2760–9770)	6405(1020–23940)	<0.01	5500(2760–9770)	6350(1020–23940)	<0.01
Creatinine (mg/dL)	0.73(0.41–7.01)	0.75(0–2.6)	<0.01	0.73(0.41–6.73)	0.74(0–1.56)	0.04
AST (U/L)	18(8–75)	18(4–166)	0.14	18(8–75)	18(4–94)	0.14
ALT (U/L)	16(7–167)	14(0–218)	<0.01	16(7–167)	14(5–100)	0.10

a *P* - values were derived using the Wilcoxon signed rank test.

Data are the median (25–75 percentile range).

b The mean blood pressure (mmHg) was calculated using the systolic blood pressure (SBP) and diastolic blood pressure (DBP) as follows: (SBP+2XDBP)/3.

Abbreviation: WBC, white blood cell; AST, aspartate aminotransferase; ALT, alanine aminotransferase.

### Characteristics of colorectal neoplasms

Table 3 describes the stage and location of the tumours in the CRC group before after matching. Categorization of patients according to cancer stage at first diagnosis revealed that 15.49% (n = 77) of patients were stage I, 24.55% (n = 122) were stage II, 25.15% (n = 125) were stage III, and 34.81% (n = 173) were stage IV before matching. After propensity score matching, 16.58% (n = 33) of patients were stage I, 24.12% (n = 48) were stage II, 23.12% (n = 46) were stage III, and 36.18% (n = 72) were stage IV. Of these, 276 (55.53%) patients had a tumour in the colon and 221 (44.47%) had a tumour in the rectum before matching and 113 (56.78%) patients had a tumour in the colon, and 86 patients (43.22%) had a tumour in the rectum after matching.

**Table 3:** Clinical features of the colorectal cancer patients after propensity score matching.

		No.	%
Before propensity score matching			
Stage of tumour	I	77	15.49
	II	122	24.55
	III	125	25.15
	IV	173	34.81
Location	Colon	276	55.53
	Sigmoid	138	27.77
	Ascending	87	17.51
	Transverse	20	4.02
	Descending	31	6.24
	Rectum	221	44.47
After propensity score matching			
Stage of tumour	I	33	16.58
	II	48	24.12
	III	46	23.12
	IV	72	36.18
Location	Colon	113	56.78
	Sigmoid	50	25.13
	Ascending	39	19.60
	Transverse	9	4.52
	Descending	15	7.54
	Rectum	86	43.22

### The prevalence of CRC based on visceral fat area tertiles

**Figure 1:**
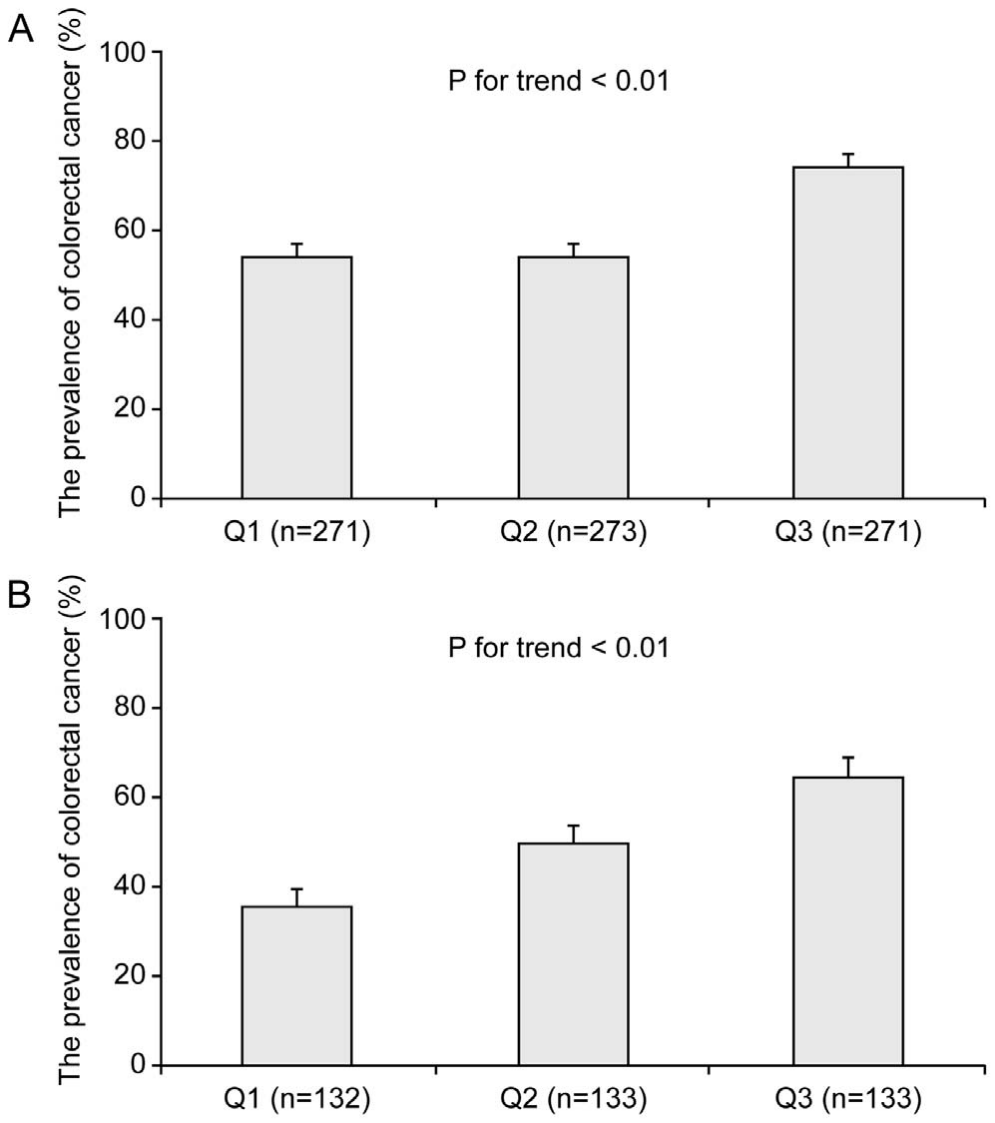
Comparison of the prevalence of colorectal cancer according to visceral fat tertiles before propensity score matching (Figure1-(A)). Comparison of the prevalence of colorectal cancer according to visceral fat tertiles after propensity score matching (Figure1-(B)). P-value was derived using the Cochran-Armitage trend test.

The prevalence values of CRC based on the 3 visceral fat area tertiles (Q1, Q2, and Q3) were shown in Figure 1. Before matching, the prevalence values of CRC based on the 3 visceral fat area tertiles (Q1, Q2, and Q3) were 54.24%, 54.21%, and 74.54%, respectively (*P*<0.01, Figure 1A). After matching the prevalence of CRC increased significantly according to the visceral fat tertiles. (30.77%, 45.76% and 69.49%, respectively (*P*<0.01). (Figure 1B)

Table 4 and 5 shows the odds ratio of the prevalence of CRC based on the visceral fat area tertiles before and after propensity score matching. The multivariate-adjusted odds ratio (95% CI) for the highest versus the lowest visceral fat tertiles were 1.80 (1.19–2.91) (unmatched) and 2.96 (1.38–6.33) (matched) after adjusting for subcutaneous fat area, mean blood pressure, WBC counts, fasting glucose, total cholesterol, creatinine, AST, and ALT levels. These positive associations persisted even after separating the prevalence of cancer into colon (OR 3.47, CI; 1.24–9.68) or rectum (OR 4.15, CI; 1.05–16.34) sites in propensity score matching group. These positive associations also persisted after separating the group according to cancer stage as stage I, II (OR 3.64, CI; 1.41–9.39) and stage III, IV (OR 3.80, CI; 1.39–10.40) in propensity score matching group.

**Table 4:** Odds ratios and 95% confidence intervals for the prevalence of colorectal cancer according to visceral fat area tertiles before propensity score matching.

	Visceral fat area tertiles, OR (95% CI)		
Model	Q1 (19.30–67.01) N = 271	Q2 (67.10–96.26) N = 273	Q3 (96.30–256.20) N = 271
Colorectal cancer			
Model 1a	1.00	1.01(0.71–1.40)	2.47(1.72–3.55)
Model 2b	1.00	0.82(0.55–1.23)	1.80(1.12–2.91)
Colon cancer			
Model 1a	1.00	0.80(0.54–1.19)	2.09(1.39–3.13)
Model 2b	1.00	0.66(0.41–1.06)	1.46(0.84–2.52)
Rectal cancer			
Model 1a	1.00	1.34(0.87–2.06)	3.13(2.00–4.88)
Model 2b	1.00	1.13(0.68–1.88)	2.18(1.22–3.91)
Stage I, II			
Model 1a	1.00	0.88(0.56–1.37)	2.58(1.66–4.01)
Model 2b	1.00	0.61(0.37–1.04)	1.59(0.89–2.83)
Stage III, IV			
Model 1a	1.00	1.08(0.74–1.59)	2.40(1.60–3.59)
Model 2b	1.00	1.04(0.65–1.67)	1.85(1.06–3.21)

**Table 5:** Odds ratios and 95% confidence intervals for the prevalence of colorectal cancer according to visceral fat area tertiles after propensity score matching.

	Visceral fat area tertiles, OR (95% CI)		
Model	Q1 (19.30–67.98) N = 132	Q2 (68.00–93.23) N = 133	Q3 (93.30–246.60) N = 133
Colorectal cancer			
Model 1a	1.00	1.86 (1.10–3.13)	3.47 (2.01–5.98)
Model 2b	1.00	1.60 (0.81–3.14)	2.96 (1.38–6.33)
Colon cancer			
Model 1a	1.00	1.43 (0.74–2.74)	3.58 (1.71–7.50)
Model 2b	1.00	1.09 (0.45–2.60)	3.47 (1.24–9.68)
Rectal cancer			
Model 1a	1.00	2.90 (1.19–7.11)	3.76 (1.60–8.83)
Model 2b	1.00	3.54 (0.97–12.91)	4.15 (1.05–16.34)
Stage I, II			
Model 1a	1.00	1.34 (0.40–4.41)	2.70 (0.62–11.63)
Model 2b	1.00	2.82 (1.19–6.67)	3.64 (1.41–9.39)
Stage III, IV			
Model 1a	1.00	1.40 (0.71–2.74)	3.32 (1.70–6.48)
Model 2b	1.00	1.93 (0.77–4.89)	3.80 (1.39–10.40)

OR: odds ratio; CI: confidence interval.

a unadjusted.

b adjusted for subcutaneous fat area, mean blood pressure, fasting glucose, total cholesterol, creatinine, aspartate aminotransferase (AST), and alanine aminotransferase (ALT) and white blood cell (WBC) counts.

OR (95% CI) were derived using conditional logistic regression test.

## Discussion

Our cross-sectional study revealed a positive relationship between abdominal visceral obesity and CRC in Korean women. Visceral fat areas in the third tertile were associated with an approximately three times higher prevalence of CRC compared with areas in the first tertile after propensity score matching and adjusting for confounding factors (odds ratio: 2.96; 95% CI: 1.38–6.33). Furthermore, this association persisted after separating the cancer sites and stages.

The prevalence of CRC has rapidly increased in the past 20 years in conjunction with the increasing prevalence of obesity worldwide [3]. Obesity is known to increase the risk of CRC significantly [10], [17] and is also related with poor prognosis after treatment [18]. Recent studies have demonstrated the important role of visceral adiposity rather than general obesity in colorectal carcinogenesis [7]–[9]. However, these studies assessed CRC risk through direct measurement of visceral fat area using CT and provided conflicting results. Recent clinical studies have shown a significant association between CRC and visceral fat area. [11], [12]. However, opposing results have also been reported. [13]. A small sample size, the confounding effect of unequal clinical characteristics of the participants, and the effect of tumour-related weight loss prior to the measurement of visceral fat are the factors that likely contributed to these unexpected results. In the present study, all of the participants underwent colonoscopy in the same hospital, and demographic characteristics between the control and CRC groups were carefully matched to reduce the effect of potential confounding factors. To our knowledge, this is the first study to compare the association between the prevalence of CRC and visceral fat area in confounding characteristics-matched cohorts.

The precise mechanisms that explain the relationship between visceral adiposity and CRC remain unclear. However, we suggest some possible mechanisms based on our results. First, visceral adipocyte-secreted proinflammatory cytokines and adipokines may induce a protumourigenic status. Chronic inflammation promotes carcinogenesis by several mechanisms, including the enhancement of cancer cell proliferation and angiogenesis [19]. Previous studies have shown that visceral adipocytes secrete higher levels of proinflammatory cytokines, including interleukin 6 (IL-6) and tumour necrosis factor-alpha (TNF-α) [20]. Increased levels of these cytokines induce a protumourigenic environment [21]. Altered adipokine secretion may also affect colorectal carcinogenesis. For example, adiponectin which exhibits anti-tumour characteristics through anti-inflammatory and proapoptotic actions [22] shows a negative correlation with visceral fat mass [23]. Furthermore, lower adiponectin levels have been reported in CRC patients [24], [25]. Therefore, systemic chronic inflammation and altered metabolic function may serve as a link for the association between visceral obesity and CRC.

Insulin resistance is another factor that supports the association between visceral obesity and CRC. The correlation between visceral adipose tissue and insulin resistance is well established [26]. Lipolysis is more active in visceral adipose tissue than in subcutaneous adipose tissue, which results in the insulin resistance status being characterized as hyperinsulinemia [27]. Hyperinsulinemia is known to increase the risk of cancers, including CRC [28], and the prevalence of CRC is higher in Type II DM patients [29]. Insulin directly stimulates colorectal carcinogenesis by activating the anti-apoptotic and mitogenic cellular signalling pathways [22]. Furthermore, the role of insulin in regulating insulin-like growth factor (IGF) axis activity is also related with the tumourigenic effect of insulin. Chronic hyperinsulinemia inhibits the production of IGF-binding protein 1 (IGFBP-1) and IGFBP-2, which results in the increased bioavailability of IGF-1 [30]. IGF-1 acts as a procarcinogen by enhancing tumour cell proliferation and decreasing cell death [31]. These results collectively suggest that the increased insulin resistance induced by visceral adiposity may be associated with an increased risk of CRC.

In addition, the direct effect of visceral adiposity on the development of CRC also should be considered. Recently, Huffman et al. demonstrated the effect of visceral fat on the development of intestinal tumours, independent of known metabolic mediators [32]. Surgical removal of the visceral fat mass significantly reduced the risk of intestinal cancer in female mice; however, it failed to increase the levels of adiponectin and reduce the level of glucose, leptin, chemokines, and total adiposity. This result suggests that visceral adiposity, at least in part, might directly affect carcinogenesis in the gastrointestinal (GI) tract, independent of insulin resistance or inflammatory adipocytokines. Further experimental studies are needed to elucidate the precise mechanism by which visceral adiposity affects the prevalence of CRC.

Our study demonstrated a significant relationship between visceral obesity and CRC in females in contrast to previous findings that showed relatively weak or no relationship between CRC and visceral obesity in female group [33]–[35]. However these studies have some limitations that most studies did not adjust the menopausal status and hormone replacement status that may affect the relationship between visceral obesity and CRC. For example Tobias et al [8]. have reported a significant relationship between CRC risk and the waist-hip ratio only in postmenopausal women who had not used HRT compared to HRT users. Because our data were obtained from postmenopausal women without HRT, our results may reflect the association of visceral obesity and CRC after minimizing the countering beneficial effects of exogenous oestrogen replacement. Additionally, many previous studies have shown a significant association between the risk of CRC and body composition, including waist circumference and waist: hip ratio in males [8], [36]. Therefore, although we only investigated the relationship between CRC and visceral obesity in females, it is possible that these significant relationships also exist in the male population. Large-scale prospective studies are required to examine the precise roles of gender in relation to cancer prevalence and visceral obesity.

Our study has several limitations. First, the cross-sectional design cannot establish a causal relationship between CRC and visceral fat area. Although our hypothesis suggested that visceral obesity might induce a higher risk of CRC, further prospective interventional studies are needed to elucidate this relationship. Second, we studied a small number of women who visited a single hospital. Therefore, our results do not allow for a generalization of the population at large. Third, we could not compare the levels of proinflammatory cytokines and adipokines that may act as important mediating factors because we used the data from the patients who visited the hospital for health check-up or for preoperative measurement. However, our results showed significantly higher WBC counts in the CRC group compared with the control group, which reflect the systemic inflammatory status of the CRC cells. Finally, due to the retrograde data collection method, clinically important variables, such as socio-economic status (including education and household income), could not be adjusted and may affect our results.

In conclusion, our results demonstrate that visceral adiposity is independently associated with the prevalence of CRC in Korean women. Although we could not determine causality, our results collectively suggest that visceral obesity, as well as total obesity, may be associated with the risk of CRC. Further interventional prospective studies with larger sample sizes are required to understand the causal relationship between visceral adiposity and the prevalence of CRC, as well as to determine the benefits of controlling visceral obesity for reducing CRC risk.
